# Adolescent internet use predicts higher levels of generalized and social anxiety symptoms for girls but not boys

**DOI:** 10.1016/j.pmedr.2023.102471

**Published:** 2023-10-13

**Authors:** Gabriel A. Tiraboschi, Gabrielle Garon-Carrier, Jonathan Smith, Caroline Fitzpatrick

**Affiliations:** a Department d’enseignement au préscolaire et au primaire, Université de Sherbrooke, 2500 Bd de l'Université, Sherbrooke, Quebec, QC J1K 2R1, Canada; b Département de psychoéducation, Université de Sherbrooke, 2500 Bd de l'Université, Sherbrooke, Quebec, QC J1K 2R1, Canada

**Keywords:** Screen media use, Adolescent internet use, Generalized anxiety, Social anxiety, Cross-lagged panel model, Sex differences

## Abstract

Past research suggests that internet use can increase the risks of internalizing symptoms in adolescents. However, bidirectional relationships between adolescent internet use and anxiety symptoms have received very little attention. Furthermore, few studies have examined these links according to sex. The present study attempts to fill this gap by investigating longitudinal associations between Canadian boys’ and girls’ internet use and symptoms of generalized anxiety and social anxiety using data from the Quebec longitudinal Study of Child Development. A sample of 1324 adolescents (698 girls, 626 boys) self-reported the number of hours per week they spent on the internet and their symptoms of generalized and social anxiety at ages 15 and 17. We estimated two cross-lagged panel models with social or generalized anxiety symptoms and internet use at age 15 predicting those same variables at age 17. Sex was used as a grouping variable and socioeconomic status was included as a control variable. Internet use at 15 predicted generalized and social anxiety symptoms at age 17 in girls, but not boys. Social and generalized anxiety symptoms at age 15 did not predict internet use at age 17 for both boys and girls. These results suggest that internet use can be a significant risk factor for the development of anxiety symptoms in adolescent girls. Girls may be more vulnerable to the negative effects of internet use due to increased sensitivity to social comparisons. Thus, helping girls develop healthier internet use habits should be a target for promoting their mental health.

## 1 Introduction

Anxiety in adolescence can be severe and long-lasting. For example, anxiety symptoms at this stage of development can increase the likelihood of experiencing emotional problems, illicit drug use, and poor academic performance (Woodward & Fergusson, 2001). Additionally, anxiety symptoms early in adolescence are the best predictors of symptom persistence and clinical diagnosis later in life (Garcia and O’Neil, 2021, Voltas et al., 2017). The majority of anxiety disorders emerge between early adolescence and young adulthood (de Lijster et al., 2017), which makes adolescence a sensitive time for the evolution of anxiety symptoms. Furthermore, studies suggest that disability may arise in cases where anxiety symptoms are not severe enough to meet the criteria for diagnosis (Angold et al., 1999).

Simultaneously, adolescent daily screen time has been rising, from 4 to 7 h before the pandemic, to 5–8 h after the pandemic, with internet use being the preferred activity (Rideout et al., 2022, Rideout and Robb, 2019). These estimates also suggest that screen time overlaps time that could be spent engaging in physical activity, face-to-face interactions, and sleeping (Hysing et al., 2015, Melkevik et al., 2010). Importantly, recent literature reviews found small but significant associations between screen media use and increased levels of internalizing symptoms and lower well-being in adolescents (Ivie et al., 2020, Keles et al., 2020, Orben, 2020, Tang et al., 2021). This link seems to be stronger for girls and internet surfing compared to other screen uses (Tang et al., 2021, Twenge and Farley, 2020).

Although studies have identified links between adolescent internet use and anxiety symptoms, the preponderance of cross-sectional designs remains an obstacle to establishing the direction of this association, as underscored in recent review articles (Keles et al., 2020, Odgers et al., 2020, Odgers and Jensen, 2020, Orben, 2020). A recent statement by the US Surgeon General recommends consistently examining the possibility of bidirectionality when examining associations between screen media use and youth mental health (U.S. Surgeon General, 2023). The reason for this is that the direction of this association should never be assumed because youth experiencing more symptoms of anxiety may choose to spend more time online as a form of avoidance or self-medication (Heffer et al., 2019, Sagioglou and Greitemeyer, 2014).

According to a recent longitudinal study, there is a prospective relationship between internet use and girls’ subsequent development of depressive symptoms between the ages of 13 and 17 (Fitzpatrick et al., 2023). However, there are few longitudinal studies that investigate the association between adolescent internet use and anxiety symptoms, and they have provided mixed messages (Boers et al., 2020, Coyne et al., 2020, Gunnell et al., 2016, Khouja et al., 2019, Zink et al., 2019). Zink et al. (2019) and Khouja et al. (2019) found that adolescent computer use predicted higher anxiety levels in later adolescence. However, Gunnell et al. (2016) and Boers et al. (2020) found cross-sectional but not longitudinal associations between internet use and anxiety symptoms. Most of those studies did not account for reverse causation.

Additionally, few longitudinal studies have considered the possibility that associations may differ between boys and girls. According to the differential susceptibility to media framework, adolescent screen use and its consequences may vary according to individual-level factors such as sex and socioeconomic status (Valkenburg & Peter, 2013). For example, girls, who tend to use the internet for more social purposes compared to boys (Ciarrochi et al., 2016), might engage more frequently in social media interactions leading to upward social comparisons, potentially affecting their self-appraisal and well-being (Appel et al., 2016, Lewallen and Behm-Morawitz, 2016, Vogel et al., 2014). Indeed, previous work has found that compared to boys, adolescent girls experience higher socio-emotional difficulties as a result of their internet use (Booker et al., 2018, Twenge and Martin, 2020). However, since most research to date has been cross-sectional, it is unknown if this sex difference would apply to one or both directions of associations between internet use and anxiety symptoms.

Moreover, few studies have disentangled internet use from other types of screen media use. Indeed, there is evidence that internet use in particular is related to internalizing symptoms (Tang et al., 2021, Twenge and Martin, 2020). Lastly, generalized and social anxiety are one of the most common mental disorders in adolescence, and their prevalence has been increasing (Creswell et al., 2020, Polanczyk et al., 2015, Tassin et al., 2014), but few studies have examined how internet use may contribute to different types of anxiety symptoms. Social and generalized anxiety, as well as other internalizing disorders, share high comorbidity, however, they significantly differ in terms of etiology, symptomatology, development, and treatment (Creswell et al., 2014, Cummings et al., 2014, Shin and Newman, 2019). Hence, they warrant independent investigations.

The present study aims to fill existing gaps in the literature by exploring the directions of the associations between adolescent internet use and the development of generalized and social anxiety symptoms, while also considering whether associations differ between boys and girls. Considering the aforementioned prior research, we hypothesize that (1) internet use will predict increased anxiety symptoms in boys and girls; (2) anxiety symptoms will predict internet use in boys and girls, (3) both directions of association would be stronger in girls.

## 2 Methods

### 2.1 Sample

The present study draws on data collected between 2013 and 2015 from the Quebec longitudinal Study of Child Development (QLSCD, 1998–2023). The QLSCD was planned and implemented by the *Institut de la statistique du Québec*. The sample originates from a randomly selected, stratified sample of 2837 infants born between 1997 and 1998 in the province of Quebec, Canada. At the study onset, 49.1 % were girls, 72 % were Canadian, and 21.7 % were below the poverty line. Participants with available estimates of socioeconomic status (SES), internet use, and anxiety symptoms for at least one of the study years were retained for our analytical sample, resulting in an analytical sample of N = 1324 for each cross-lagged panel model, with 53 % of the sample being girls (n = 698). To handle missing data in our model we selected the full information maximum likelihood method.

### 2.2 Procedure

The QLSCD was approved by the ethics review boards of the *Institut de la statistique du Québec*. Informed consent was obtained from the adolescents and parents. The variables for this study were collected in 2013 and 2015 when participants were 15 and 17 years of age, respectively. In 2013 and 2015, the QLSCD included a mental health and adjustment component. These questions were self-reported by youth using an online questionnaire and have been previously validated (Côté et al., 2017).

### 2.3 Internet use

At ages 15 and 17, youth self-reported how much time they spent per week accessing the internet on a computer playing games, doing searches, chatting, or being on Facebook (excluding time spent on the internet at school) in the last 3 months. Questionnaires are available here (https://www.jesuisjeserai.stat.gouv.qc.ca/informations_chercheurs/outils_collecte/E16-QELJ_EN.pdf). Answer options included: (1) None; (2) Less than an hour; (3) 1 to 2 h; (4) 3 to 5 h; (5) 6 to 10 h; (6) 11 to 14 h; (7) 15 to 20 h; or (8) more than 20 h. Scores were converted to continuous measures of hours per week by using the midpoint value for each possible answer option – except 20 or more hours which was scored as 20 h.

### 2.4 Anxiety measures

Youth at ages 15 and 17 self-reported generalized and social anxiety symptoms over the past 12 months, using items derived from the Mental Health and Social Inadaptation Assessment for Adolescents (questionnaires are available at www.jesuisjeserai.stat.gouv.qc.ca/informations_chercheurs/outils_collecte/outils_collecte_an.html). This tool was created to reflect symptoms included in the Diagnostic and Statistical Manual 5th edition. For each of the mental health outcomes, a mean score was created and converted to a score ranging from 0 to 10, with 10 indicating the highest level of symptomology. The following dimensions were considered as outcomes: *Generalized Anxiety Disorder* (GAD) based on 9 items, Cronbach's alpha = 0.81, and *Social Anxiety Disorder* (SAD) assessed from the 8 items, Cronbach's alpha = 0.84.

Generalized anxiety was derived from the following 9 items: “I was too fearful or nervous”; “I had worries that interfered with my everyday life”; “I worried about my past behaviour”; “I worried about my school work”; “I worried about my own health”; “I worried about my loved ones (family, friends)”; “I worried about my relationships with my friends (i.e. making and keeping friends)”; “I was concerned about my appearance or weight”; “I found it difficult to control the worry”.

Social anxiety was assessed from the 8 following items: “I was afraid of or tried to avoid situations where there would be a lot of people”; “I was afraid of or tried to avoid situations where I would have to meet a lot of new people”; “I was afraid of or tried to avoid situations where I would need to do things in front of others”; “I was afraid of or tried to avoid situations where I would need to speak up in class”; “I was afraid of or tried to avoid situations where I would need to read out loud in front of others”; “I have disliked being in situations in which I attracted attention”; “Even if I was with people I trust, I have worried about social situations that draw attention to me”; “I’ve turned red or started to shake when faced with social situations I fear”.

### 2.5 Control variable

An index of socioeconomic status was derived from mother and father reports of income, level of education, and occupational prestige (Willms & Shields, 1996).

### 2.6 Data analysis strategy

#### 2.6.1 Sex-invariance

We first examined invariance across sex. Specifically, we estimate variance/co-variance matrixes for internet use and anxiety symptoms at ages 15 and 17. We ran separate variance/co-variance matrixes for generalized and social anxiety symptoms. Fit indices were calculated from a freely estimated matrix and this result was compared to the fit indices (χ^2^) of a model with equivalent covariances across groups (i.e., constrained covariances). If the test of difference is statistically significant, this suggests that the matrixes vary significantly across groups and that we should reject the null hypothesis that boys and girls have a similar variance/co-variance structure.

#### 2.6.2 Statistical model

**Fig. 1:**
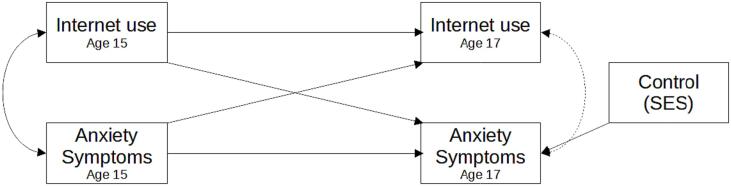
General model structure of both cross-lagged panel models used in this study that includes variables of adolescents’ mental health and internet use at ages 15 and 17 in Quebec, Canada. Note. Each separated cross-lagged panel model has this same overall structure depicted in this figure. Each rectangle represents a variable, and each straight arrow indicates a regression path. Diagonal straight arrows indicate cross-lagged paths and horizontal arrows autoregressive paths. Curved arrows indicate co-movements. The two leftmost rectangles denote the first wave of data collection at age 15 and are exogenous variables. The two rectangles to the right represent the second wave of data collection at age 17 and are the endogenous variables of the model. The rightmost rectangle is the control variable, SES. Anxiety symptoms boxes represents symptoms of either GAD or SAD, depending on the model.

We employed standard cross-lagged panel models to explore the direction of the association between internet use and anxiety symptoms. We estimated two different models, one for each type of anxiety disorder (generalized anxiety or social anxiety symptoms). In each cross-lagged panel model, internet use at age 15 and anxiety symptoms at age 15 were used as exogenous and/or predictor variables and internet use at age 17 and anxiety symptoms at age 17 were used as endogenous and/or outcome variables. For each model, there were two autoregressive paths: internet use at age 15 predicting internet use at age 17, and anxiety symptoms at age 15 predicting anxiety symptoms at age 17. The autoregressive path captures the proportion of a variable (internet use or anxiety symptoms) at baseline (age 15) that persists directly to the same variable at age 17. Our model also includes two cross-lagged paths. The first cross-lagged path represents the effect of internet use at age 15 predicting anxiety symptoms at age 17, and the second, the reverse association: anxiety symptoms at age 15 predicting internet usage two years later. The cross-lagged paths are the effects that are directly related to our first and second hypotheses, and it uses the first time point of one variable to predict the second time point of a different variable. This effect mirrors causal research, emphasizing temporal precedence akin to Granger causality principles (Zyphur et al., 2020). Additionally, our model included co-variances between the variables within the same time points. Finally, socioeconomic status was employed as a control (exogenous) variable for the anxiety symptoms outcome variable. The common structure of both CLPMs is depicted in Fig. 1.

For each of our models, we used maximum likelihood estimator with robust error calculation and Yuan-Bentler correction because we did not observe univariate normal distributions within our variables of media use. All statistical analyses were conducted in JASP (JASP Team, 2022) using lavaan syntax in SEM modules.

## 3 Results

### 3.1 Descriptive statistics

The test of invariance was significant for the generalized (Δ χ^2^ = 331, Δdf = 28, *p* <.001) and the social anxiety (Δ χ^2^ = 328.56, Δdf = 28, *p* <.001) matrices. This indicated that a sex-based analysis without equality constraints would improve the structural equation models’ fit significantly. Because of this, we used sex as a grouping variable in each of our models without any equality constraints.

Descriptive statistics are presented in Table 1. Boys and girls spent a similar amount of time on the internet when comparing their averages across ages 15 and 17 (mean = 7.38 vs 7.43 h per week, *p* =.876). Girls scored significantly higher than boys on measures of generalized (mean = 3.4 vs 4.9, *p* <.001) and social anxiety symptoms (mean = 1.8 vs 2.9, *p* <.001), which further justified conducting analyses grouping by sex.

**Table 1:** Descriptive statistics for adolescents (boys and girls) internet use and mental health symptoms between ages 15 and 17 in Quebec, Canada.

	Mean (SD)		Min-Max		N		P-value
	Girls	Boys	Girls	Boys	Girls	Boys	
*Screen time (hours/week)*							
Internet	7.38 (4.86)	7.43 (5.18)	0 – 20	0.25 – 20	607	481	0.876
*Mental health outcomes*							
GAD	4.94 (1.83)	3.41 (1.74)	0.55 – 9.16	0 – 8.89	632	516	<0.001
SAD	2.98 (2.08)	1.88 (1.74)	0 – 9.37	0 – 9.69	633	516	<0.001
*Control variable*							
SES	−0.01 (1.04)	0.02 (0.94)	−3.03 to 2.26	−3.03 to 2.26	698	626	0.806

*Note.* GAD = Generalised Anxiety Disorder. SAD = Social Anxiety Disorder. Answer options for internet usage range from less than one hour per week to a maximum of 20 h or more per week. Mental health scales ranged from 0 to 10. The values represent averages from the years 2013 and 2015. Data compiled from the final master file of the Québec Longitudinal Study of Child Development (2013–2015), ©Gouvernement du Québec, Institut de la statistique du Québec.

### 3.2 Cross-lagged panel models

The results of the cross-lagged paths from each model are summarized in Table 2 with the values of the unstandardized (B) and standardized estimates (β), their 95 % confidence intervals, and two-tailed p-values. Full results are available at the Open Science Framework at https://osf.io/ux8fm/files/osfstorage/64286957d9cfc31675b3d03f.

**Table 2:** Regression results for cross-lagged paths between internet use and anxiety symptoms from adolescents at ages 15 to 17, in Quebec Canada.

	Girls				Boys			
Parameter estimate	B	95 %CI	β	p	B	95 %CI	β	p
*Model1. GAD / Internet*								
Int 15 → GAD 17	0.025	0.003, 0.047	0.071	0.024	0.009	-0.017, 0.035	0.028	0.485
GAD 15 → Int 17	-0.030	-0.243, 0.183	-0.011	0.784	-0.048	-0.334, 0.237	-0.015	0.740
*Model 2. SAD / Internet*								
Int 15 → SAD 17	0.029	0.000, 0.057	0.070	0.048	0.000	-0.026, 0.025	-0.002	0.960
SAD 15 → Int 17	0.171	-0.034, 0.376	0.067	0.102	0.231	-0.058, 0.521	0.069	0.117

*Note.* Because displaying the full results of all models would not fit in a table, only paths relevant to our hypotheses (the cross-lagged paths) in each model are present in the table. *B* represents the unstandardized estimate of each regression path, followed by the *95 %CI* which represents the 95 % confidence interval of the unstandardized estimate. *β* represents the standardized estimate of the regression. P-values lower than 0.05 were considered significant. The numbers 15 and 17 depicts the participants’ age at the time of data collection. GAD = Generalised Anxiety Disorder symptoms. SAD = Social Anxiety Disorder symptoms. Int = internet use in a typical week. Data were compiled from the final master file of the Québec Longitudinal Study of Child Development (2013–2015), ©Gouvernement du Québec, Institut de la statistique du Québec.

#### 3.2.1 Generalized anxiety symptoms

The model examining associations between internet use and generalized anxiety symptoms between ages 15 and 17 generated excellent fit indices (RMSEA = 0.000, CFI = 1.000, and χ^2^ = 0.179, *p* =.914). For girls, internet use at age 15 predicted more generalized anxiety symptoms at age 17 (β = 0.07, *p* =.024), but generalized anxiety symptoms at 15 did not predict girls’ internet use at age 17 (β = -0.01, *p* =.784). Boys’ internet use at age 15 did not predict generalised anxiety symptoms levels at age 17 (β = 0.02, *p* =.485), and boys generalised anxiety symptoms at age 15 did not predict internet usage two years later (β = -0.01, *p* =.740). The autoregressive paths show that internet use was somewhat stable between ages 15 and 17, for both girls (β = 0.32, *p* <.001) and boys (β = 0.35, *p* <.001). The auto-regressive paths results also show that generalized anxiety symptoms were stable across the two years for both girls (β = 0.51, *p* <.001) and boys (β = 0.53, *p* <.001). Finally, lower SES predicted more generalized anxiety symptoms at age 17 for girls (β = -0.07, *p* =.042) but not for boys (β = -0.02, *p* =.468).

#### 3.2.2 Social anxiety symptoms

The model examining associations between adolescent internet use and social anxiety symptoms generated good fit indices (RMSEA = 0.000, CFI = 1.000, and χ ^2^ = 0.093, *p* =.954). For girls, internet use at age 15 predicted higher levels of social anxiety symptoms at 17 (β = 0.07, *p* =.048), however, their social anxiety symptoms at age 15 were not associated with internet use at age 17 (β = 0.06, *p* =.102). Boys’ internet use at age 15 was not associated with social anxiety symptoms at 17 (β = -0.002, *p* =.960), and their social anxiety symptoms at age 15 did not predict internet use two years later (β = 0.06, *p* =.117). Social anxiety symptoms were stable across ages 15 and 17 for both girls (β = 0.55, *p* <.001) and boys (β = 0.49, *p* <.001). SES did not predict social anxiety symptom levels at age 17 for both girls (β = -0.05, *p* =.122) and boys (β = -0.03, *p* =.402).

## 4 Discussion

In this study, we examined bidirectional associations between adolescent internet use and anxiety symptoms between the ages of 15 and 17. We also examined whether the association differed between boys and girls. We hypothesized that (1) internet use would predict anxiety symptoms, (2) anxiety symptoms would predict internet use, and (3) associations would be more pronounced in girls. Supporting our first hypothesis, we found that internet use at age 15 predicted social and generalized anxiety symptoms at age 17 for girls, with no such effect observed in boys. Our second hypothesis, suggesting that anxiety symptoms precede increased internet use, was not supported, in both boys and girls. Regarding the third hypothesis, a sex-specific pattern emerged, indicating that internet use predicted anxiety symptoms exclusively in girls. However, no significant sex differences were observed for the reverse direction of this association. Our results corroborate previous findings where adolescent computer use increased both social and generalized anxiety levels over time (Zink et al., 2019).

In our study, adolescent anxiety symptoms did not predict internet usage. In another recent study, Orben et al. (2022) found bidirectional associations between social media use and lower life satisfaction among adolescents aged 10 to 19, suggesting that bidirectional associations could have emerged if it was possible to observe a larger time frame. However, it is worth considering that adolescents with higher anxiety levels may not necessarily use more internet, contrary to hypotheses proposing that individuals perceive the internet as a coping mechanism for anxiety (Heffer et al., 2019, Sagioglou and Greitemeyer, 2014). Nonetheless, our statistical design accounts for reverse causation and temporal precedence of variables, rendering one of the closest approaches to a fully causal design when working with observational data. This highlights the quality of our findings as it bridges gaps in a predominantly cross-sectional literature, as noted in recent literature reviews (Keles et al., 2020, Odgers et al., 2020, Odgers and Jensen, 2020, Orben, 2020).

Importantly, we found that internet use predicted later changes in anxiety symptoms only in adolescent girls, corroborating previous findings of sex differences (Booker et al., 2018, Twenge and Farley, 2020, Twenge and Martin, 2020). Our results also align with the findings of Fitzpatrick et al. (2023), who observed a comparable unidirectional relationship between adolescent internet usage and subsequent depression symptoms only in girls. Social comparisons could likely explain the observed effects (Vogel et al., 2014) as girls tend to use the internet for more social purposes compared to boys (Ciarrochi et al., 2016). Adolescent girls’ engagement with social media can lead to repeated exposure to curated glimpses of their peers’ and friends’ lives (Schlosser, 2020). This may then create an environment of upward social comparison (Lewallen and Behm-Morawitz, 2016, Vogel et al., 2015) where adolescents compare their own social lives and physical appearance to what is being presented by their peers on the screen. These unfavorable comparisons may lead to doubt and worry and have the potential to affect adolescents’ social self-appraisal and well-being (Appel et al., 2016).

In addition to facing social comparisons, prolonged experiences with social media can lead to other negative experiences, such as the fear of missing out or feeling left out (Beyens et al., 2016), loneliness, (Barry et al., 2017), and cyberbullying (Zhu et al., 2021). These negative experiences may lead to avoidance and worries surrounding social situations and may increase symptoms of social anxiety. Furthermore, these experiences may exacerbate adolescent’s generalized anxiety symptoms, by decreasing self-esteem and triggering worrying about safety, economic status, and level of competence.

More time spent online can also increase adolescent girls’ exposure to content that is objectifying and unrealistic which can be detrimental to youth body image (Rodgers & Melioli, 2016). Exposure to objectifying images of women rather than men is more common and is likely to have a more profound influence on the self-esteem of girls (Vandenbosch & Eggermont, 2012), which can help explain why the effect of internet use was present only in girls for both generalized and social anxiety symptoms. Finally, girls have a higher prevalence of anxiety symptoms than boys (Lewinsohn et al., 1998, Racine et al., 2021) which could render them more vulnerable to the anxiogenic effects of internet use.

Our study presents limitations. First, our measure of internet use provided only limited information on youth activities. For instance, it would be helpful for future studies to consider how adolescent internet use for communication and non-communication purposes may differentially impact adolescent anxiety (Selfhout et al., 2009). Additionally, our measure of internet use did not account for adolescents’ internet use on their phones and our measures of anxiety and internet use were self-reported, which can lead to social and memory bias as well as shared measurement bias. Finally, in the present study we were unable to consider potential mediators of the observed associations. For example, adolescents’ relationship quality with peers and friends could be explored in future studies.

In conclusion, our results suggest that increased internet use is a risk factor for the development of anxiety symptoms in adolescent girls. As a modifiable risk factor, youth media habits may therefore represent an important intervention target. However, this longitudinal association still needs to be further explored. For example, future research could clarify whether this effect of internet use reflects comorbidity between different symptom clusters, and directly compare its effect on specific types of anxiety symptoms. Additionally, because standard cross-lagged panel models aggregate within- and between-person sources of variance, this could be addressed in future research.

## CRediT authorship contribution statement

**Gabriel A. Tiraboschi:** Conceptualization, Methodology, Formal analysis, Writing – original draft, Visualization. **Gabrielle Garon-Carrier:** Resources, Writing – review & editing, Funding acquisition. **Jonathan Smith:** Writing – review & editing. **Caroline Fitzpatrick:** Conceptualization, Resources, Data curation, Writing – review & editing, Supervision, Funding acquisition.

## Declaration of Competing Interest

The authors declare that they have no known competing financial interests or personal relationships that could have appeared to influence the work reported in this paper.

## Data Availability

The authors do not have permission to share data.
